# Exploring Brazilian Immigrant Mothers’ Beliefs and Practices Regarding Their Preschool Children’s Oral Health: A Qualitative Study Conducted in the United States

**DOI:** 10.3390/ijerph21121574

**Published:** 2024-11-27

**Authors:** Denise Lima Nogueira, Maria Gabriela Miranda Fontenele, Adriana Bento, Steven A. Cohen, Mary L. Greaney, Ana Cristina Lindsay

**Affiliations:** 1 Department of Nursing, Faculty Luciano Feijão, Sobral 62050-215, Ceará, Brazil; deniseln2009@hotmail.com; 2 Faculty of Nursing, Federal University of Ceará, Fortaleza 60355-636, Ceará, Brazil; maria.gabriela129@hotmail.com; 3 Brazilian Community Partner, University of Massachusetts Boston, Boston, MA 02125, USA; adrianabentousa@gmail.com; 4 Department of Public Health, College of Health Sciences, University of Rhode Island, Kingston, RI 02881, USA; steven_cohen@uri.edu (S.A.C.); mgreaney@uri.edu (M.L.G.); 5 Department of Urban Public Health, Robert and Donna Manning College of Nursing and Health Sciences, University of Massachusetts Boston, Boston, MA 02125, USA

**Keywords:** oral health, Brazilian immigrant mothers, preschool children, health belief model, qualitative study

## Abstract

Parents’ beliefs and practices significantly shape young children’s oral health (OH), particularly during preschool years when these habits are being established. Immigrant parents often face challenges in promoting OH due to cultural, financial, and logistical barriers. This qualitative study explored OH beliefs, practices, and barriers among Brazilian immigrant mothers in the United States (U.S.). Semi-structured interviews, guided by the Health Belief Model (HBM), were conducted with mothers of children aged 2–5 years. Thematic analysis, also guided by the HBM, revealed four key themes: (1) beliefs about children’s OH; (2) awareness of the parent’s role in fostering early OH behaviors; (3) identification of risk and protective factors affecting children’s OH; and (4) perceived barriers to maintaining children’s optimal OH. Mothers emphasized the importance of proper oral hygiene, regular dental check-ups, and balanced diets for promoting children’s OH. Despite recognizing their role in supporting oral hygiene routines and care, mothers noted barriers such as limited access to care, linguistic barriers, and difficulty adopting and maintaining healthy OH practices due to work and family demands. Notably, 96.6% (*n* = 28) of the mothers scored low on the SASH scale (SASH < 2.99), reflecting low acculturation levels, which may further contribute to these challenges. Study findings highlight the critical role of parental beliefs and practices in shaping early OH behaviors and the unique challenges faced by Brazilian immigrant mothers. Culturally relevant public health interventions, including tailored education and improved access to affordable dental care, are essential for enhancing OH outcomes in young children from these families.

## 1. Introduction

Oral health (OH) in early childhood is crucial to overall health and development, influencing children’s immediate wellbeing and long-term health outcomes [1]. Establishing healthy oral habits at a young age helps prevent common dental issues like cavities and gum disease, which are particularly prevalent among children [2]. Parents play a central role in shaping their children’s OH behaviors [3,4,5,6,7]. Parents’ OH-related behaviors are influenced by various factors, including parents’ socioeconomic and demographic characteristics, acculturation levels, and biological, behavioral and psychosocial factors [3,8,9]. Research indicates that less acculturated Hispanic/Latinx parents often have lower OH knowledge and face greater challenges in accessing dental care for their children compared to parents who are more acculturated [8,10,11]. These differences are largely due to differences in sociocultural factors between the home and host countries (e.g., availability of dentists, familiarity with the dental care system, and OH education). They are not solely attributed to acculturation levels [12]. Therefore, it is important to consider and understand the impact of cultural differences on OH knowledge and practices as they play a significant role in shaping health behaviors [8,9,10,13,14,15].

Immigrant populations in the United States (U.S.), including Brazilian immigrants, often face distinct challenges in maintaining their children’s OH due to socioeconomic, cultural, and financial factors [12,13,14]. Despite being a rapidly growing population group, there is a lack of research specifically addressing the OH of children in Brazilian immigrant families in the U.S. This research is needed to explore the unique beliefs and challenges influencing OH practices within this population [16,17]. Understanding the perspectives of Brazilian immigrant mothers, who often take primary responsibility for their children’s healthcare, is crucial for developing targeted public health interventions [18,19].

The Health Belief Model (HBM) provides a valuable framework for understanding how beliefs influence health behaviors [20,21]. The theory posits that individuals’ health actions are influenced by their perceptions of susceptibility to a health problem, the severity of the potential health outcome, the benefits of preventive action, and the barriers to taking that action [20,21]. In OH, these dimensions can help elucidate how parents perceive and manage their children’s OH and dental care, particularly within immigrant communities where cultural and economic factors may significantly impact health behaviors [22,23].

Guided by the HBM, the current study explored Brazilian immigrant mothers’ beliefs and practices related to their children’s OH to understand the factors that shape behaviors and outcomes in this population. By analyzing narratives around children’s OH, we aim to identify key factors influencing mothers’ management of oral hygiene and dental care, including their awareness of risk and protective factors, their role in fostering healthy behaviors, and the barriers they face in maintaining optimal OH practices for their children [24,25]. This research will provide a foundation for future research and intervention strategies tailored to this community’s needs.

## 2. Materials and Methods

### 2.1. Study Design

Given the lack of existing studies, qualitative research methods are particularly well suited as an initial step in exploring the maternal beliefs and practices surrounding children’s OH in Brazilian immigrant families [22,23]. Qualitative approaches allow for a nuanced understanding of the experiences and cultural contexts that influence health behaviors, providing valuable insights that quantitative methods may overlook [22,23].

This qualitative study used in-depth interviews to explore the beliefs and practices of Brazilian immigrant mothers regarding their preschool-aged children’s oral health. Qualitative methods are particularly well suited for this research as they capture rich, detailed narratives and offer insights into the cultural and contextual factors that influence health behaviors [26].

### 2.2. Participant Selection

The study focuses on mothers of children aged 2 to 5 years, a critical period for establishing OOH habits. Participants were purposively selected to ensure a range of experiences and perspectives among Brazilian immigrant mothers in the U.S. The inclusion criteria were as follows: (1) being a Brazilian immigrant mother, (2) being 21+ years old, (3) having at least one child aged 2–5 years, and (4) residing in the U.S. for at least six months. The last criterion was selected to capture the experiences of mothers who have had some exposure to the U.S. healthcare system and cultural environment.

Flyers outlining the study purpose and eligibility were posted in community organizations and local businesses in urban and suburban areas with significant Brazilian immigrant populations within the Greater Boston region, including Framingham, Somerville, Everett, Chelsea, Fall River, Lowell, Brockton, and Worcester [16]. These areas are attractive to Brazilian immigrants due to various cultural, social, and economic factors. Individuals interested in participating contacted the research team via the phone number provided on the flyer for more information and to express their interest.

In addition to flyer recruitment, participants were also recruited through social media, personal networks, and referrals via snowball sampling [27,28]. Interested individuals reached out by phone or directly engaged with research staff, who then conducted eligibility screening and scheduled interviews.

### 2.3. Data Collection

At the start of each scheduled interview, conducted via Zoom, participants were informed about the study’s purpose, procedures, and their rights, including the right to withdraw at any time without penalty. Informed consent was obtained electronically before proceeding with the interviewer-administered questionnaire. Participants then completed the brief questionnaire, followed by the in-depth qualitative interview.

The brief survey collected sociodemographic information (e.g., age, marital status, educational attainment, annual household income, number of children between 2 and 5 years of age, primary language spoken at home, length of residence in the U.S., etc.) and acculturation level. Acculturation was measured using the Short Acculturation Scale for Hispanics (SASH), a 12-item scale validated for Latino populations [29]. The SASH assesses language use, media use, and ethnic social relations, and has good reliability (Cronbach’s alpha reliabilities 0.92–0.89 for the overall SASH scale, 0.89 for language use, 0.88 for media preference, and 0.72 for ethnic and social relations) [29,30]. An acculturation score was computed for each participant by averaging across 12 items, measured on a scale of 1 to 5. The scale developers recommend an average of 2.99 as the cut-point; scores equal to or above this point represent higher levels of acculturation, and scores below this point represent lower levels of acculturation [29].

Immediately after the brief questionnaire was completed, participants then took part in an in-depth interview, also via Zoom. This video conferencing platform allowed for face-to-face interaction while ensuring participant convenience and safety. Zoom enabled mothers to join the interview from their preferred locations, minimizing barriers such as transportation and childcare.

To ensure rigor and credibility, the research team included members with expertise in qualitative research and a strong understanding of the participants’ cultural context [31]. The survey and interviews were conducted in Brazilian Portuguese, the participants’ native language, by two native Brazilian researchers with specialized knowledge in qualitative methods and experience working with immigrant populations. Both researchers are fluent in Portuguese and have strong ties to the Brazilian community. One researcher (ACL) is a Brazilian immigrant to the U.S. and has lived in areas where participants reside, providing valuable insights into the challenges Brazilian immigrants face. Professionally, one researcher is a non-practicing dentist (ACL), and the other is a nurse (DLN). Both interviewers have postgraduate training in public health and maternal and child health. These combined cultural and professional backgrounds contribute to a deeper understanding of OH disparities and family health practices, enabling respectful and empathetic engagement with participants.

The interviews lasted approximately 60 min and were audio-recorded with participants’ consent. Data collection was guided by a semi-structured interview guide informed by the HBM and the existing literature [20,21]. The interview guide was pilot tested to refine questions and ensure clarity. The interview guide explored mothers’ beliefs and practices regarding their children’s OH [20,21,32,33]. The guide included open-ended questions to elicit detailed narratives about participants’ perceptions of OH, perceived risks, severity and benefits, and barriers to maintaining optimal OH. Questions also explored mothers’ role in fostering healthy oral habits and their experiences accessing dental care in the U.S. Data collection continued until saturation of themes was achieved, meaning no new information or themes emerged from the interviews.

### 2.4. Data Analysis

The Zoom interviews were audio-recorded and professionally transcribed verbatim in Brazilian Portuguese. The transcripts were reviewed, de-identified, and analyzed in Portuguese using thematic analysis informed by the HBM [20,21,34,35]. A codebook was developed using the HBM as a framework for coding and analysis [20,21,34,35]. Transcripts were systematically coded, and new codes were added to the codebook as they emerged during analysis. The analysis was conducted using MAXQDA [36], a qualitative data analysis software that supported systematic coding, organization, and text retrieval. Two bilingual researchers [DLN, ACL] independently coded the transcripts using MAXQDA [34,35,36], discussing any discrepancies and reaching consensus to ensure accuracy in the coding process. The analysis followed an iterative process, with codes being reviewed multiple times by the research team to identify emerging themes and patterns [34,35]. Thematic analysis facilitated the identification of both explicit and implicit meanings in the data, offering a comprehensive understanding of the factors influencing children’s OH behaviors among Brazilian immigrant mothers [35]. Consistency and coding validity were maintained through regular team discussions and cross-checking of codes.

The coders addressed reflexivity by maintaining detailed field notes and reflecting on their potential biases and their impact on data collection and analysis. The research team conducted regular debriefing sessions to discuss and mitigate biases, ensuring that the findings accurately represented the participants’ perspectives [35].

### 2.5. Ethical Considerations

The Institutional Review Board (IRB) of the University of Massachusetts Boston approved the study. All participants were assured of the confidentiality and anonymity of their responses. Personal identifiers were removed from the transcripts, and audio recordings were securely stored and accessible only to the research team. Participants were given an incentive (USD 40.00) to compensate for their participation in the study.

## 3. Results

### 3.1. Sociodemographics

The study sample consisted of 29 participants, with a mean age of 33.9 years (SD = 6.5). The majority identified as mixed race (55.2%), followed by White (31.0%) and Black (13.8%). Most participants were married or living with a partner (75.9%). About half (55.2%) of participants completed high school (Table 1). Regarding household income, 27.6% earned less than $45,000 per year. Most participants (86.2%) had one child between the ages of 2–5 years, and all were born in Brazil, predominantly from the state of Minas Gerais (69.0%).

Participants reported living in the U.S. for an average of 5.8 years, with 96.6% having scores < 2.99 on the SASH scale, indicating low acculturation. All participants spoke Portuguese at home. Most (93.1%) had public/government-sponsored healthcare insurance, and over half (55.2%) had dental care insurance through MassHealth, the MA Medicaid program, which provides health insurance coverage to low-income individuals and families (Table 1).

### 3.2. Themes

The analysis revealed four key themes: (1) beliefs about children’s OH; (2) awareness of the parent’s role in fostering early OH behaviors; (3) identification of risk and protective factors affecting children’s OH; and (4) perceived barriers to maintaining children’s optimal OH.

Mapping the identified themes to the HBM, beliefs about children’s OH and awareness of parents’ role in children’s early OH behaviors correspond to the construct perceived susceptibility and perceived benefits. The theme identification of risk and protective factors for children’s OH aligns with the dimensions of perceived severity and perceived benefits. The theme perceived barriers to optimal OH relates to the construct perceived barriers.

#### 3.2.1. Beliefs About Children’s OH

Mothers defined good OH as proper care and hygiene of the mouth, absence of dental issues, regular check-ups, and ensuring the development of healthy teeth. One mother explained, “*Good oral health for me means not having bad breath, tooth pain, or cavities*” (Mother M#5). Another added, “*Healthy teeth without cavities, good hygiene of both teeth and tongue, and good nutrition will lead to good oral health*” (M#8). They associated good OH with attentive parental care, including overseeing daily brushing, serving a balanced diet low in sugary and ultra-processed foods, and assisting and supervising children’s daily tooth brushing and hygiene. Mothers emphasized oral hygiene, preventive care, and proper nutrition as key components of OH.

In contrast, mothers linked poor OH in childhood to poor oral hygiene routines (e.g., inconsistent tooth brushing), unhealthy dietary habits, and lack of regular dental check-ups. One mother shared, “*Poor brushing, not going to the dentist, eating too many sweets*” (M#10). Another added, “*Many children at his age already have cavities because of too many sweets... too much sugar. Those front teeth, yellowish*” (M#5).

When reflecting on their children’s oral health, most mothers reported that it was generally good, citing the absence of dental problems and the use of preventive care. However, some expressed concerns like bad breath or occasional dental issues. One mother said, “*Her oral health is perfect, no inflammation, her teeth are fine*” (M#11). Another noted, *“[…] sometimes he wakes up with bad breath, so I don’t know. It can be strong, so I’m concerned about his oral health*” (M#7). Some mothers expressed confidence in their current practices. “*For now, I don’t see a problem*” (M#3), one said, reflecting confidence in her child’s OH.

Some mothers also noted the prevalence of poor OH among Brazilian families in the U.S., largely due to difficulty accessing pediatric dental care. For example, one mother stated, “*[…] Our girl’s teeth got bad because we couldn’t find care. Dentists here are expensive and hard to get appointments with*” (M#6). Another mother shared, “*I’ve heard friends complain about tooth pain, but it’s hard to make an appointment or it takes too long*” (M#7).

#### 3.2.2. Awareness of Parent’s Role in Fostering Children’s Early OH Behaviors

The analysis revealed that mothers recognize their pivotal role in shaping their children’s early oral health behaviors. They believe that guiding children in proper oral hygiene practices—such as demonstrating brushing techniques, encouraging regular brushing, and modeling these behaviors—is key to fostering good oral health. For example, one mother explained, “*The key is to do it with them, brush your teeth and teach them, and explain why*” (M#26), while another said, *”[...] encouraging tooth brushing, seeking the dentist for follow-up, flossing*” (M#3). One mother also emphasized making tooth brushing a fun routine: “*The child sees, is watching... you turn it into a game, try other ways, and then the example and the fact that you persist every day until it becomes a habit for the child”* (M#28).

Mothers also highlighted the importance of controlling their children’s intake of sugary and ultra-processed foods, providing on-going guidance on oral hygiene, and ensuring regular dental check-ups. One mother shared, “*In terms of diet, control what your children eat, especially regarding snacks, candy, sweets [...]. And encourage them to clean their mouth, remember to brush before bed, and before going to school in the morning*” (M#1). Another mother added, “*At least once a year, take them to the dentist, showing them educational programs that help. Yes, brushing their teeth, eating healthy*” (M#4).

#### 3.2.3. Identification of Risk and Protective Factors Affecting Children’s OH

Mothers identified key behaviors and several risk factors affecting their children’s OH, including insufficient tooth brushing, improper oral hygiene practices, excessive consumption of sugary and ultra-processed foods, lack of regular dental check-ups, and insufficient parental supervision. They also highlighted the care provided by others, such as daycare staff, preschool teachers, and family members, as a potential risk for poor oral hygiene.

One mother noted, “*If the child eats a lot of sweets, doesn’t brush teeth often, doesn’t go to the dentist, those are the risk factors*” (M#5). Another mother echoed similar concerns, stating, “*Children who have a lot of candy in their diet, who have poor brushing habits, and who don’t go to the dentist regularly*” (M#10). Additionally, concerns about the care provided by others were expressed by a mother who remarked, “*Because with others, we don’t know what their diet is like, their day-to-day life. We never know*” (M#17).

On the other hand, mothers cited several protective factors for promoting optimal OH. For instance, one mother emphasized, “*Parents brush the child’s teeth regularly, use fluoride toothpaste, provide healthy food, and go to the dentist frequently*” (M#4). Another mother stressed, “*Good oral hygiene from a young age, regular dental visits, and a proper diet—avoiding too many sweets—are essential*” (M#11).

#### 3.2.4. Perceived Barriers to Maintaining Optimal OH

The analysis identified several barriers Brazilian immigrant mothers face regarding their children’s OH. One common barrier was the lack of time and fatigue from work, which led to inconsistency in brushing routines. Mothers described how long work hours and physical exhaustion prevented them from consistently supervising their children’s oral hygiene. For instance, one mother expressed, “*There are days when I get home [...] and I don’t give him a toothbrush because I’m too tired*” (M#5). Another mother said, “*I think here we work a lot; we get really tired. There are days when I get home, take a shower, and go brush my teeth, and he comes after me, and I don’t give him a toothbrush because I’m too tired [...]*”. (M#8).

Another important barrier was the intensity of daily routines, where busy schedules often resulted in rushed or poorly executed tooth brushing. Some mothers admitted that their hectic lifestyles limited their ability to monitor their children’s oral hygiene as closely as they would like, with one mother stating, “*Sometimes it’s a rush [...] you end up brushing their teeth a little poorly*” (M#2). In contrast, some mothers believed that daily routines and work activities were not barriers to providing adequate OH care for their children. For example, one mother said, “*I’ll be honest, I don’t think so. I have time to help them, there’s healthy food at home, I take them to the dentist, so, like, no*” (M#2). Another mother echoed this sentiment, “*I don’t think so because even if they stayed all day, in the morning, I would be brushing their teeth and at night before they go to bed, which is what dentists recommend—brushing at least twice a day*” (M#23).

Additionally, mothers spoke of children’s resistance and lack of cooperation in brushing their teeth as being challenging. Many mothers noted that daily tooth brushing became a “daily struggle”, with children often refusing to cooperate. One mother shared her frustration: “*I have to hold him every now and then to do it, because he won’t let me*” (M#3). Another mother stated, *“[...] it’s very hard for me to brush her teeth; she doesn’t let me. So, she ends up wanting to brush on her own, but she doesn’t do it right*”. (M#10).

Mothers also encountered difficulty accessing dental services, exacerbated by logistical issues such as lack of transportation or scheduling conflicts. One mother mentioned, “*I‘ve seen friends complaining to me, my son has a toothache, so he needs to make an appointment, but it’s difficult. It’s either hard making an appointment, or it’s taking a long time*” (M#7). Another mother mentioned, “*I depend on the father [...], and usually when the dentist is scheduled for him, it’s always in the morning when my husband is at work*” (M#24), highlighting the impact of family schedules on attending dental appointments.

In cases where other caregivers (e.g., preschool teachers, daycare center staff, family members, and nannies) were involved in childcare, mothers worried about losing control over their children’s diet and oral hygiene practices. One mother commented, “*we don’t have control over what is in front of our eyes, so we can’t avoid other people giving them certain kinds of food*” (M#4), referring to the food choices made by other caregivers (M#4). Similarly, some mothers were concerned about the lack of encouragement for brushing in daycare settings, with mothers noting that schools did not promote regular brushing after meals. One mother shared, “*[…] because at school, they don’t have the habit of brushing the teeth. I asked him, he told me that there they don’t brush the teeth*” (M#12).

Financial constraints related to dental care were another significant barrier. Some mothers pointed out that U.S. health insurance plans did not cover certain dental treatments, making access to care more difficult. One participant remarked, “*the issue of insurance [...] limits this*” (M#29). Furthermore, the high cost of dental treatments was a deterrent, with participants highlighting the expense of dental care in the U.S. Another mother commented, “*[…] the cost of living is very high, and dentists are very expensive*” (M#15).

Linguistic and cultural differences presented additional barriers, particularly for mothers who were not fluent in English. They reported struggling to navigate the healthcare system, communicate with dental professionals, and ensure their children received adequate care. For example, one mother stated, “*I’m not fluent in English [...] I need to find a place where [...] they will treat him well and help me in my language*” (M#13). Another mother shared, “*[...] that day I went, he (referring to the dentist) only spoke Spanish, and sometimes he (the dentist) gets a little impatient with us when we don’t understand things*” (M#17).

Lastly, a key barrier was a lack of knowledge about dental service organizations, particularly among more recent immigrants. Mothers expressed uncertainty about how the dental care system worked in the U.S. or whether insurance covers services. One mother shared, “*I don’t know how to do it here or if I’ll have to pay to take them to the dentist*” (M#22). Another mother said, “*...there are many mothers who don’t have the guidance to take care of their children. They don’t know what’s important. There are also many mothers who haven’t managed to take their child to a dentist yet because they don’t know where to find dental care for their children...*” (M#12). This lack of familiarity often delayed or prevented access to essential dental services for their children.

## 4. Discussion

This study, guided by the HBM [20,21], provides valuable insights into Brazilian immigrant mothers’ perceptions and practices related to their children’s OH and access to dental care. To our knowledge, this is the first study specifically examining Brazilian immigrant mothers in the U.S., making it a significant contribution to understanding OH within this growing, minoritized immigrant population [16]. The analysis found that while mothers are generally proactive in managing their children’s OH, several key themes emerged, highlighting opportunities to enhance both OH outcomes and access to dental care for children in Brazilian immigrant families.

### 4.1. Parental Beliefs and Roles

The findings emphasize the crucial role of parental beliefs and awareness in shaping early OH behaviors. Mothers consistently viewed their children’s OH as encompassing proper oral care, hygiene, regular dental check-ups, and preventive measures. These beliefs align with the HBM’s dimensions of perceived susceptibility and perceived benefits, suggesting that mothers who engage in preventive care practices are more likely to perceive their children as having good OH [21]. Similar observations were reported in previous studies, which found that parents with higher OH literacy were more likely to engage in preventive dental care for their children [37,38].

However, despite these positive beliefs and practices, some mothers expressed concerns about their children’s OH, especially when facing barriers to maintaining optimal OH. This underscores the need for on-going support and education to address these concerns and reinforce the importance of preventive care. Research by Walker et al. [39] highlights that education and resources tailored to parental knowledge can improve children’s OH outcomes. Additionally, low acculturation levels in immigrant populations may limit access to these educational resources, underscoring the importance of culturally and linguistically appropriate outreach [40,41,42,43,44].

### 4.2. Risk and Protective Factors

The study identified several risk factors that mothers perceive as affecting children’s OH, including inadequate tooth brushing, high consumption of sugary and ultra-processed foods, lack of regular dental check-ups, and childcare provided by other caregivers. These findings align with the HBM’s construct perceived susceptibility, emphasizing the importance of addressing these risk factors through targeted interventions. Protective factors, such as consistent and proper oral hygiene practices and regular dental visits, were critical to maintaining optimal OH. This is consistent with findings by Tiwari, Wilson et al. [8] and Finlayson, Beltran, and Becerra [40], who identified similar barriers and protective factors among Latino families, emphasizing the need for public health initiatives that promote these protective behaviors and address the identified risk factors.

Moreover, acculturation likely plays a role in shaping these behaviors, as lower acculturation levels may result in immigrant parents relying on traditional health practices or facing barriers such as language difficulties when seeking dental care [41,43,44]. This reinforces the need for public health initiatives that promote protective behaviors and tailor interventions to address not only socioeconomic barriers but also the specific cultural and linguistic challenges faced by less acculturated families [44,45].

### 4.3. Challenges and Barriers

An important finding is the identification of barriers mothers face to maintaining their children’s optimal OH, including lack of time for adequate OH, difficulties accessing dental care, and financial constraints. These barriers reflect real-world obstacles impacting the consistency and effectiveness of OH practices. Previous studies [7,39,46] have noted that logistical challenges, such as limited access to dental care and financial constraints, significantly affect OH practices among Latino parents. Although most participants in our study reported having dental insurance through MassHealth, access to these services is often limited in underserved urban areas with large immigrant populations, such as those represented in our study.

Low acculturation levels may further exacerbate challenges accessing dental care due to limited English proficiency or unfamiliarity with the U.S. healthcare system [43,44]. Study findings suggest that daily routines and work activities should be seen as barriers and opportunities where support and strategies can be developed to enhance OH care. Public health interventions must address these challenges by improving access to dental care and providing resources to manage time and financial constraints effectively. For example, dental practices could extend their hours to accommodate working parents’ schedules and offer interpreting services to overcome language barriers. Additionally, it is important to develop culturally tailored interventions that address the specific barriers faced by immigrant families, particularly those with lower acculturation levels. These interventions should target multiple levels of the social ecological model—intrapersonal/individual, interpersonal, institutional/organizational, community, and policy—to ensure a comprehensive approach [47]. Previous studies underscore the significance of such multi-level approaches in effectively overcoming these challenges [10,13,46,48,49,50].

### 4.4. Implications for Public Health Interventions

The insights gained from this study highlight the need for public health interventions tailored to the specific challenges faced by Brazilian immigrant parents [45,46]. Addressing barriers such as access to dental care and financial constraints can significantly impact the effectiveness of OH programs. For instance, Finlayson, Beltran, and Becerra [40] and Rai and Tiwari [6] suggest that targeted interventions to improve dental care access and culturally tailored education can help overcome these barriers. Additionally, reinforcing the role of parents in promoting early OH behaviors and providing support to overcome practical challenges are essential components of successful interventions.

An important consideration is the low acculturation levels in this sample of Brazilian immigrant mothers, which can have important effects on OH behaviors and access to care [13,14,15]. Mothers with low acculturation may face language barriers, making it difficult to communicate with healthcare providers or understand the healthcare system. This ultimately limits their ability to seek timely and appropriate dental care for their children [10,13,14,15]. Limited familiarity with the U.S. healthcare system can also create confusion about navigating dental insurance, appointment scheduling, or understanding the importance of preventive dental visits [44,45,46]. These challenges are further compounded by differing cultural beliefs about dental care, which may lead to reliance on traditional practices or underestimation of the importance of preventive OH measures [40,41,42,43,44].

Culturally and linguistically appropriate interventions are essential to address these issues effectively [10,46,47]. Building trust between healthcare providers and immigrant families, particularly those with lower acculturation levels, is crucial for encouraging regular dental visits and early interventions. This can be facilitated by providing interpreting services in dental practices, ensuring that non-English-speaking parents can communicate effectively and feel confident in their children’s care [43,44]. Additionally, dental practices should consider offering extended service hours to accommodate working parents who may struggle to find time for appointments during regular business hours.

Tailored health literacy programs, provided in the native language of the parents (Brazilian Portuguese) can further empower them to take an active role in maintaining their children’s OH [8,10,37,49]. Such programs could focus on increasing awareness of the importance of preventive dental care and providing practical information on navigating the U.S. healthcare system. These interventions can help mitigate the possible effects of low acculturation by fostering better understanding and engagement with OH services. Tiwari, Wilson, and Kabani [8] and Kabani, Elangovan, and Ramesh [10] also highlight the importance of addressing acculturation effects, which can influence OH behaviors and access to care among Latino parents. Similar approaches should be adopted for Brazilian immigrant families, recognizing that acculturation significantly shapes health behaviors and outcomes.

Public health initiatives should be designed with a deep understanding of the unique challenges faced by immigrant families and offer comprehensive support to bridge the gap between their current practices and the optimal preventive care needed to ensure their children’s long-term OH. Programs like WIC (Women, Infants, and Children) offer a valuable platform for integrating OH education as they provide an opportunity to educate parents on preventive care, navigating the healthcare system, and fostering acculturation in ways that promote better OH outcomes. WIC, a federal program designed to support low-income pregnant, breastfeeding, or postpartum women and infants and children under five, aims to improve nutrition and increase access to essential health services. By incorporating OH discussions into WIC’s nutrition education and services, it can further reduce health disparities and support families in achieving better overall health, including OH.

## 5. Conclusions

The findings from this study underscore the critical importance of parental beliefs, awareness, and proactive involvement in maintaining their children’s OH. The analysis reveals that mothers perceive their role as pivotal in fostering optimal OH through guidance, modeling, and regular dental care. There is a need for public health interventions specifically designed to address the unique challenges faced by Brazilian immigrant parents with low levels of acculturation, particularly in accessing dental services and managing dietary influences.

In conclusion, this study demonstrates that effectively addressing identified risk factors, reinforcing protective behaviors, and overcoming barriers are essential to public health strategies. Notably, acculturation significantly impacts these dynamics, influencing access to care and the adoption of health practices [10]. Tailored interventions are needed, and should consider cultural and linguistic challenges, especially for less acculturated families, for supporting parents and ensuring optimal OH for children, particularly in immigrant communities.

Moreover, it is essential to recognize the social determinants of oral health (OH), which include the broader contextual factors affecting minority and immigrant populations. Elements such as socioeconomic status, education, neighborhood environment, and access to healthcare resources significantly shape OH outcomes [48]. By understanding and addressing these determinants, public health initiatives can be designed more effectively to target the root causes of health disparities. This comprehensive approach not only enhances access to care but also empowers families to make informed decisions about their children’s OH.

By aligning public health efforts with the specific needs of Brazilian immigrant families and acknowledging the impact of social determinants, we can improve OH outcomes and promote long-term wellbeing. Future research should continue to explore the intersection of acculturation, parental beliefs, and OH practices, as well as the social determinants that influence these dynamics, to develop targeted strategies that effectively address the needs of this growing immigrant population group.

## 6. Limitations and Strengths

The findings of this study should be interpreted with consideration of study limitations. Specifically, selection bias may affect the results, as the sampling methods could overrepresent individuals who are more engaged or hold stronger opinions. Additionally, the context-specific nature of the study means that its findings may not be generalizable to other immigrant groups. Social desirability bias could also influence the accuracy of reported behaviors, and the reliability of thematic analysis is subject to subjective interpretation despite efforts to ensure consistency.

Nevertheless, this study has notable strengths. Semi-structured interviews facilitated a rich, in-depth exploration of participants’ perspectives, capturing nuanced details about their experiences and cultural influences on OH. Furthermore, the purposive and snowball sampling strategies contributed to diverse experiences, offering a comprehensive view of the participants’ beliefs and practices. By focusing on Brazilian immigrants, the study addresses a critical gap in the literature and provides culturally relevant data that enhances our understanding of this specific population.

## Figures and Tables

**Table 1:** The sociodemographic and cultural characteristics of the sample (N = 29).

Variables	Mean	SD	N	%
Age	33.9	6.5	29	100
Race				
White			9	31.0
Black			4	13.8
Mixed race (Pardo/mestizo)			16	55.2
Marital status				
Married/Living with Partner			22	75.9
Divorced/Separated			3	10.3
Single			4	13.8
Educational attainment				
Less than high school diploma			4	13.8
High school graduate			16	55.2
More than high school			9	31.0
Household income				
<USD 45,000/year			8	27.6
≥USD 45,000/year–<USD 65,000			14	48.3
≥USD 65,000/year			7	24.1
Number of children between 2 and 5 years				
1			25	86.2
2			4	13.8
Born in Brazil				
Yes			29	100
States of origin				
Minas Gerais			20	69.0
São Paulo			2	6.9
Espírito Santo			3	10.2
Paraná			2	6.9
Amazonas			1	3.5
Rio Grande do Norte			1	3.5
Years of Residence in the U.S.				
<5 years			14	48.3
≥5 years–<10 years			12	41.4
≥10 years			3	10.3
Primary language spoken at home				
Portuguese			29	100
SASH				
<2.99			28	96.6
≥2.99			1	3.4
Healthcare insurance				
Public/government-sponsored			27	93.1
Private			2	6.9
Dental Care Insurance				
Yes (MassHealth)			16	55.2
No			13	44.8

## Data Availability

The data underlying this study are not publicly available due to confidentiality agreements with participants. However, reasonable requests for data may be made to the corresponding author, and these will be considered on a case-by-case basis, in compliance with ethical and legal restrictions.
